# Multiple Means to the Same End: The Genetic Basis of Acquired Stress Resistance in Yeast

**DOI:** 10.1371/journal.pgen.1002353

**Published:** 2011-11-10

**Authors:** David B. Berry, Qiaoning Guan, James Hose, Suraiya Haroon, Marinella Gebbia, Lawrence E. Heisler, Corey Nislow, Guri Giaever, Audrey P. Gasch

**Affiliations:** 1 Laboratory of Genetics, University of Wisconsin–Madison, Madison, Wisconsin, United States of America; 2 Terrance Donnelly Centre for Cellular and Biomolecular Research, Toronto, Canada; 3 Department of Pharmaceutical Sciences, University of Toronto, Toronto, Canada; 4 Banting and Best Department of Medical Research, University of Toronto, Toronto, Canada; 5 Genome Center of Wisconsin, University of Wisconsin–Madison, Madison, Wisconsin, United States of America; The University of North Carolina at Chapel Hill, United States of America

## Abstract

In nature, stressful environments often occur in combination or close succession, and thus the ability to prepare for impending stress likely provides a significant fitness advantage. Organisms exposed to a mild dose of stress can become tolerant to what would otherwise be a lethal dose of subsequent stress; however, the mechanism of this acquired stress tolerance is poorly understood. To explore this, we exposed the yeast gene-deletion libraries, which interrogate all essential and non-essential genes, to successive stress treatments and identified genes necessary for acquiring subsequent stress resistance. Cells were exposed to one of three different mild stress pretreatments (salt, DTT, or heat shock) and then challenged with a severe dose of hydrogen peroxide (H_2_O_2_). Surprisingly, there was little overlap in the genes required for acquisition of H_2_O_2_ tolerance after different mild-stress pretreatments, revealing distinct mechanisms of surviving H_2_O_2_ in each case. Integrative network analysis of these results with respect to protein–protein interactions, synthetic–genetic interactions, and functional annotations identified many processes not previously linked to H_2_O_2_ tolerance. We tested and present several models that explain the lack of overlap in genes required for H_2_O_2_ tolerance after each of the three pretreatments. Together, this work shows that acquired tolerance to the same severe stress occurs by different mechanisms depending on prior cellular experiences, underscoring the context-dependent nature of stress tolerance.

## Author Summary

Cells experience stressful conditions in the real world that can threaten physiology. Therefore, organisms have evolved intricate defense systems to protect themselves against environmental stress. Many organisms can increase their stress tolerance at the first sign of a problem through a phenomenon called acquired stress resistance: when pre-exposed to a mild dose of one stress, cells can become super-tolerant to subsequent stresses that would kill unprepared cells. This response is observed in many organisms, from bacteria to plants to humans, and has application in human health and disease treatment; however, its mechanism remains poorly understood. We used yeast as a model to identify genes important for acquired resistance to severe oxidative stress after pretreatment with three different mild stresses (osmotic, heat, or reductive shock). Surprisingly, there was little overlap in the genes required to survive the same severe stress after each pretreatment. This reveals that the mechanism of acquiring tolerance to the same severe stress occurs through different routes depending on the mild stressor. We leveraged available datasets of physical and genetic interaction networks to address the mechanism and regulation of stress tolerance. We find that acquired stress resistance is a unique phenotype that can uncover new insights into stress biology.

## Introduction

All organisms must respond to stressful stimuli that result from external environmental changes or internal defects caused by mutation and disease. Decades of research have characterized the mechanisms for surviving individual stresses, by mapping downstream protection systems as well as upstream signaling pathways that mediate these responses [1]–[7]. However, much less is known about the effects of combinatorial stress treatments and how cells defend against compound stresses. For example, stressful environmental changes in nature likely occur together, either simultaneously or in close succession, especially for microbes living in natural conditions. How the mechanisms of stress defense differ when cells experience successive stresses rather than a single insult is poorly understood.

Successive stress treatments can cause cells to acquire resistance to a severe (‘secondary’) stress after experiencing an initial mild (‘primary’) dose of stress. Acquired stress resistance can occur if the mild and severe treatments represent the same stressor but also across different mild and severe stresses (known as ‘cross-stress’ protection). Acquired stress resistance has been observed in diverse organisms, including yeast, bacteria, archaea, plants, flies, and mammals including mice and humans [8]–[20]. A better understanding of how cells are able to increase their resistance to further insults has potential medical application for decreasing cell death and improving human recovery from stressful events such as chemotherapy treatments and ischemia following heart attack or stroke [21]–[23].

In yeast, it had been suggested that acquired stress resistance in general, and cross-stress protection specifically, may be due to activation of the Environmental Stress Response (ESR) [24]–[30]. The ESR is a gene expression response commonly activated by a wide variety of stressful conditions [24], [25]. It includes induced expression of ∼300 genes involved in stress defense, and reduced expression of ∼600 genes broadly involved in protein synthesis and growth. However, we previously showed that ESR activation alone is insufficient to explain cross-stress protection [31]. Moreover, the ‘general-stress’ transcription factors *MSN2* and *MSN4* are conditionally required for acquired stress resistance, depending on the precise combination of mild and severe stress treatments [31]. These results revealed that the mechanism of acquired stress resistance is more complex than previously suspected and suggested that the response occurs through different mechanisms depending on the mild stress pretreatment.

Many studies have identified genes required to survive a single dose of oxidative stress, and several studies characterized increased tolerance after preconditioning (reviewed in [5], [6], [32]). The majority of these studies used single-gene approaches, though several used the yeast deletion collection to interrogate the entire genome [33]–[36]. Kelley *et al*. (2009) identified genes required to survive an acute dose of H_2_O_2_ and genes necessary to acquire H_2_O_2_ resistance following a mild H_2_O_2_ pretreatment. They found that the genes required for acquisition of H_2_O_2_ tolerance only partially overlapped the genes required to survive the acute dose alone, indicating that the mechanism of acquired H_2_O_2_ tolerance is distinct from the mechanism of basal H_2_O_2_ resistance [34]. The mechanism of cross-stress protection, in which the mild pretreatment is a different stressor than the subsequent severe stress, is largely unexplored.

Here, we leveraged the power of yeast genetics and high-throughput analysis to identify genes and processes important for acquired resistance to severe H_2_O_2_ stress after each of three mild pretreatments (mild NaCl, heat shock, or DTT treatment). We used the pooled yeast deletion collection [37], [38], including ∼4,800 homozygous diploid nonessential genes (homozygous profiling), ∼1,300 heterozygous diploid essential genes (haploinsufficiency profiling), and 1,140 strains harboring DAmP alleles of the essential genes (in which the transcript is destabilized due to insertion of a drug marker into the 3' UTR [39]) to query the vast majority of the yeast genome in a single experiment. We found that, although each pretreatment provided similar levels of subsequent H_2_O_2_ resistance, different genes and processes were required depending on the mild stress used. Functional analysis of the genes required during each pretreatment provided new insights into the relationships between regulators and processes. Acquired stress resistance thus serves as a unique phenotype through which to uncover new insights into stress biology.

## Results

### Methods summary

**Figure 1:**
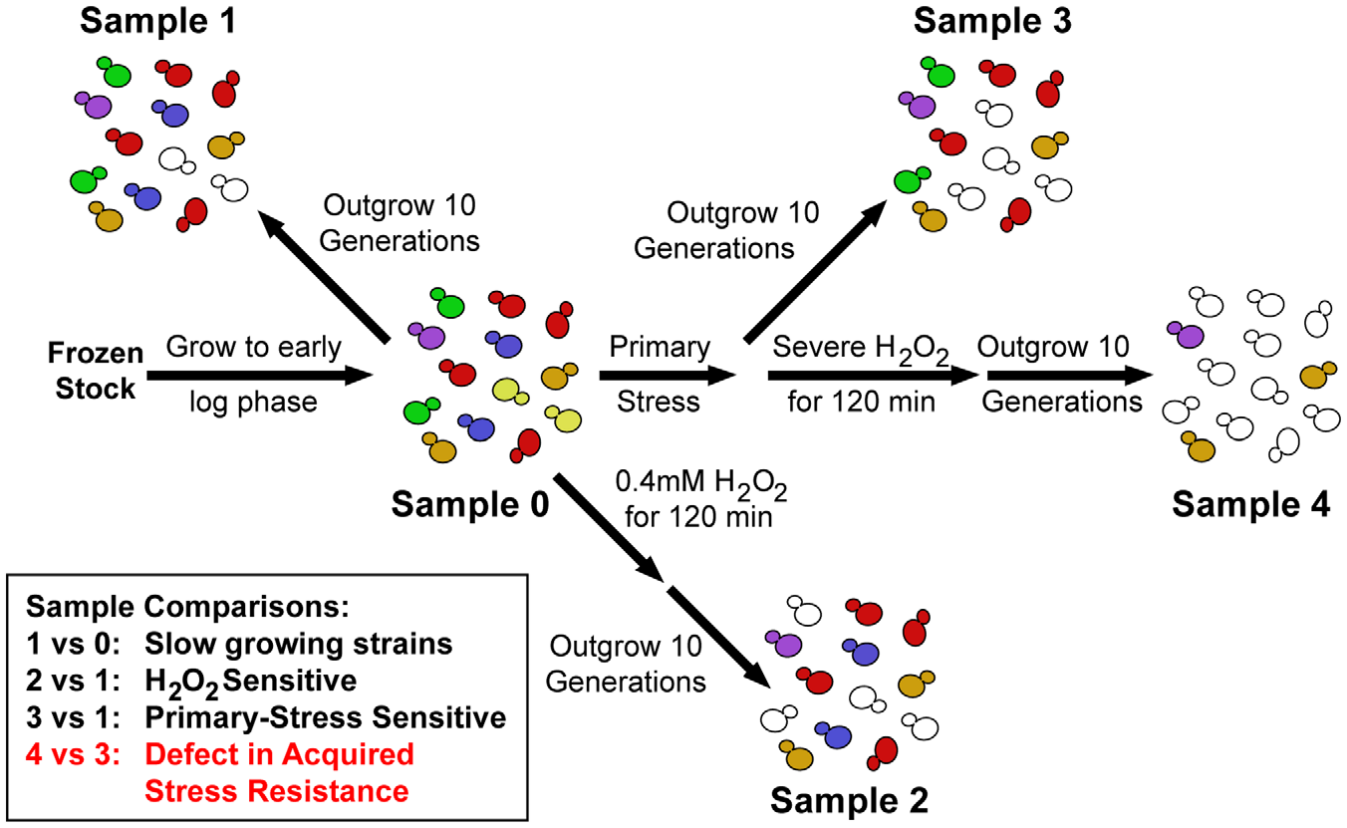
Experimental overview. Pooled mutant libraries were grown >7 generations in log phase (Sample 0) before being exposed to one of three mild (‘Primary’) stress pretreatments. Cells were then either outgrown 10 generations (Sample 3) or washed and exposed to severe H_2_O_2_ for 2 hours followed by 10 generations outgrowth (Sample 4). Strains sensitive to a mild dose of H_2_O_2_ were identified in a separate control experiment (Sample 2). Strains of interest were determined through the sample comparisons listed (see text for details).

We exposed the pooled yeast deletion libraries [37]–[39] to severe doses of H_2_O_2_ after pretreatment with one of three mild stresses (Figure 1). These mild stresses were chosen because they each produce increased H_2_O_2_ tolerance in wild type but present different initial challenges to the cell. The pooled library was exposed to either 60 min of 0.7 M NaCl, 60 min at 40C after a 30°C–40°C heat shock, or 2 h exposure to 2.5 mM DTT - each treatment produces roughly equivalent levels of subsequent H_2_O_2_ tolerance in wild-type cells. After the pretreatment, cells from the culture were washed and then exposed for 2 hours to either 1.0 mM or 1.2 mM H_2_O_2_. Exposure to these H_2_O_2_ doses kills >85% of untreated wild-type cells but results in >80% viability in cells previously exposed to mild stress (data not shown). To identify mutant strains with defects in acquired H_2_O_2_ tolerance, an aliquot of the pooled library was removed from stress at each sample point (Figure 1) and outgrown for precisely 10 generations to dilute dead cells from the population. Relative strain abundances were then measured by quantifying the unique ‘barcode’ sequences (identified by microarray and/or deep sequencing analysis). A defect in acquired H_2_O_2_ tolerance was identified based on the log_2_ change in strain abundance before and after treatments (see Figure 1 and Materials and Methods for details). We also identified 202 strains that were sensitive to a low dose of 0.4 mM H_2_O_2_ in the absence of any pretreatment (*e.g.* Sample 2 versus Sample 1, false discovery rate (FDR)<0.05, Table S1); these included many genes and regulators known to be important for the H_2_O_2_ response [33]–[35]. Because we were interested only in genes important for the acquisition of stress tolerance, we removed from consideration strains with equal fitness defects at both the low and ‘secondary’ doses of H_2_O_2_ and strains sensitive to the mild stress treatment alone (identified by comparing Sample 3 versus Sample 1). Strains that met all of these criteria in replicate experiments were defined as having a specific defect in acquiring resistance to H_2_O_2_.

### Little overlap in genes required for acquisition of stress resistance after each pretreatment

A substantial fraction of the yeast genome was required for acquisition of normal H_2_O_2_ resistance after at least one of the three pretreatments. In all, 841 strains (∼13% of measured genes) displayed a defect in acquiring H_2_O_2_ tolerance, with 225 strains identified following mild NaCl treatment, 308 after heat shock, and 497 after DTT treatment (Table S2). Validation experiments were performed for 48 strains, the majority of which were predicted to have a defect after one or more pretreatments and three that were predicted to have no defect after any pretreatment. We measured mutant phenotypes in response to all three mild stresses, allowing us to quantify false positive and false negative rates, by competing each identified strain or the isogenic wild type against a GFP-marked strain (see Materials and Methods for details). This defined an upper limit of ∼25% false positives and ∼25% false negatives; however, these values are almost certainly inflated, because our validation assay does not precisely mirror the selection experiments and was performed using the haploid deletion library. Nonetheless, the results validate that the majority of our strain identifications are accurate.

**Figure 2:**
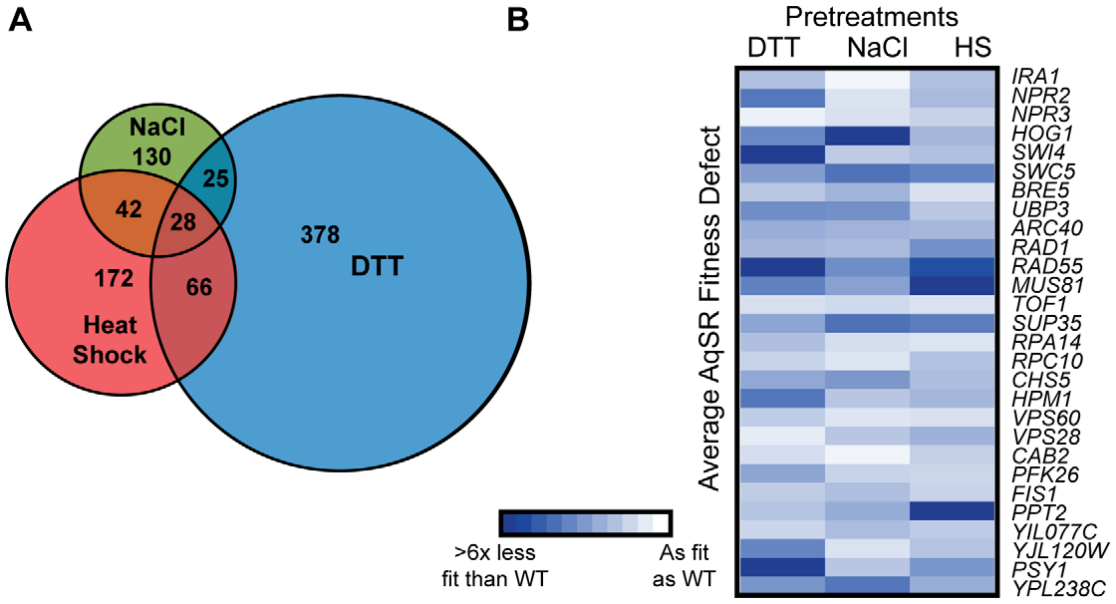
Genes important for acquired H_2_O_2_ resistance after each pretreatment. (A) The number of genes necessary for acquiring H_2_O_2_ resistance after NaCl, heat shock, or DTT treatment are shown in the Venn diagram, with shared genes identified in the overlap. (B) The average fitness defect following exposure to 1.0 mM H_2_O_2_ is shown for 28 strains that had a defect after all three pretreatments. Each row represents an individual strain, and each column represents a single pretreatment. Average fitness defects in acquired stress resistance are represented by increased color intensity, according to the key. Data shown are for deletion strains, with the exception of three genes (*ARC40*, *CAB2*, and *YPL238C*) for which fitness scores were taken from strains expressing the DAmP alleles. Gene annotations are found in Table 1.

There was surprisingly little overlap between the genes necessary for acquired H_2_O_2_ resistance following each mild stress (Figure 2A) – only 28 strains had defects following all three primary-stress conditions (Table 1). This observation cannot be explained by the nominally high false-negative rate: of the 48 strains validated, 34 were predicted to have conditional defects - only two of these 34 (6%) proved to have a universal defect in the validation experiments. There was also low overlap between the genes necessary following these primary stresses compared to genes required after mild H_2_O_2_ pretreatment [34]. There is little functionality in common to the 28 shared genes, with a few exceptions. There were several genes involved DNA damage repair and vacuolar processes, along with negative regulators of Ras (*IRA1*) and TOR (*NPR2* and *NPR3*) signaling, which themselves suppress the stress response (reviewed in [4], [40]). However, even among the 28 strains with universal defects, the magnitude of their fitness defects varied dramatically depending on the initial mild stress used (Figure 2B). Thus, even the genes necessary in all three cases were not equally important following each mild-stress pretreatment.

**Table 1:** Genes required following all 3 pretreatments.

IRA1	Negatively Regulates Ras
NPR2	Negatively Regulates TORC1
NPR3	Negatively Regulates TORC1
HOG1	MAP Kinase, HOG Pathway
SWI4	Component of SBF Complex
SWC5	Component of SWR1 Complex
BRE5	Ubiquitin protease cofactor
UBP3	Ubiquitin specific protease
ARC40	Subunit of ARP2/3 Complex
RAD1	DNA Repair
RAD55	DNA Repair
MUS81	DNA Repair
TOF1	Sister chromatid cohesion after DNA damage; unknown function
SUP35	Translation Termination Factor
RPA14	Subunit of RNA Polymerase I
RPC10	Subunit of RNA Polymerase I, II, and III
CHS5	Component of exomer complex
HPM1	AdoMet-dependent methyltransferase
VPS60	Late endosome to vacuole transport
VPS28	Component of ESCRT-I Complex
CAB2	Coenzyme A Biosynthesis
PFK26	6-phosphofructo-2-kinase
FIS1	Mitochondrial fission
PPT2	Phosphopantetheine:protein transferase
YIL077C	Unknown Function
YJL120W	Dubious ORF, unknown function
PSY1	Dubious ORF, unknown function
YPL238C	Dubious ORF, unknown function

The 28 genes with a defect in acquired H_2_O_2_ resistance following each of the three pretreatments, with abbreviated functional annotations.

We also found limited overlap in functional processes enriched in each group of required genes (Table 2). Genes necessary for acquired H_2_O_2_ tolerance after NaCl pretreatment were involved in proteolysis as well as HOG signaling, a pathway well known to respond to NaCl. By contrast, genes important following heat shock were enriched for DNA damage repair, protein transport, and late endosome to vacuole transport. Functions enriched in the group of the DTT-required genes included ubiquitin-dependent and -independent protein catabolism, ribosomal proteins, and regulation of translation. Other processes were shared for two of the three mild stressors (Table 2). Considering this and the above results, we conclude that genes and processes necessary to acquire H_2_O_2_ resistance are largely distinct and determined by each pretreatment.

**Table 2:** Functional enrichments in genes necessary to acquire H_2_O_2_ tolerance.

Primary Stress	Functional Category	p-value
NaCl	Proteolysis	5.31E-05
	HOG signaling	1.01E-06
Heat Shock	DNA repair	1.24E-08
	Protein transport	1.13E-06
	Ubiquitin-dependent protein catabolism	3.20E-07
	Multivesicular body sorting pathway	
	Late endosome to vacuole transport	4.54E-05
	Ubiquitin-independent protein catabolism	2.20E-12
DTT	Ubiquitin-dependent protein catabolism	5.35E-09
	Regulation of translation	3.32E-08
	Ribosomal proteins	2.70E-07
NaCl-HS Overlap	Negative regulation of Ras signaling	4.04E-05
NaCl-DTT Overlap	Ubiquitin-dependent protein catabolism	1.22E-06
HS-DTT Overlap	Protein targeting to vacuole	8.94E-05

Functional categories significantly enriched among the genes necessary for acquisition of H_2_O_2_ resistance following each mild pretreatment or shared between pairs of mild stresses are shown. P-values were calculated using a hypergeometric distribution and are significant after Bonferroni correction.

### Low correlation between fitness contribution and gene expression

Previous studies showed little correlation between a gene's expression change during stress and its requirement to survive prolonged treatment with that stressor [37], [41]–[46]. However, we and others showed that gene expression changes are not required to survive the initial stress treatment, but rather are critical for acquired resistance to the secondary stress [31], [47]–[49]. We therefore wondered if gene expression changes were more correlated with genes' involvement in acquired, rather than basal, stress tolerance. However, we too found low correlation between a gene's fitness effect and its expression change during the mild-stress treatment. Roughly 24% of genes necessary for acquired H_2_O_2_ tolerance after mild NaCl or heat shock were induced in expression during pretreatment (a slight enrichment above that expected by chance, *p = *0.048). In fact, genes necessary for acquired H_2_O_2_ tolerance after DTT treatment were actually enriched for DTT-repressed genes (*p* = 0.0003). Conversely, the majority of genes whose expression increased during each mild stress treatment played no role in subsequent H_2_O_2_ tolerance. Thus, gene induction is a poor predictor of gene requirement for both basal [37] and acquired stress tolerance (see Discussion).

Initiation of the yeast ESR was originally proposed to give rise to cross-stress protection [24]–[30]; however, we showed that initiation of the ESR cannot explain acquired stress resistance [31]. Consistent with this notion, we observed little enrichment of ESR genes in any of the gene lists identified above (with the exception of repressed-ESR genes among those required after DTT pretreatment). While individual ESR genes can contribute substantially to the acquisition of stress tolerance (see below), the ESR as a whole seems not to be the sole determinant of the resistance acquired.

The results above indicate that acquired H_2_O_2_ tolerance occurs through distinct modes, rather than a common mechanism, for each mild-stress pretreatment. We were interested in exploring the possible reasons for the low overlap in required genes. Below we present example cases of three models that explain the low overlap in required genes.

### Condition-specific regulators are only required during specific pretreatments

One possibility is that different upstream signaling pathways mediate the cellular response, even if the downstream effectors of acquired H_2_O_2_ tolerance may be the same across pretreatments. Indeed, an example of condition-specific signaling is seen if NaCl is the pretreatment. Several transcriptional regulators and signaling molecules were important for acquired H_2_O_2_ resistance after NaCl stress, including the stress-activated transcription factor *MSN2* [25]–[27], [50] and the majority of HOG signaling components (including *HOG1*, *PBS2*, *SSK2*, *SSK1*, *STE50*, and *CDC42*) (Figure S1). Notably, none of the corresponding deletion strains was sensitive to a 1 h exposure to 0.7 M NaCl (data not shown), but all had major defects in acquired H_2_O_2_ tolerance. The Hog1 pathway regulates expression of stress-responsive genes specifically during osmotic shock and related stresses but not other conditions (J. Clarke and APG, unpublished data). Consistently, none of the HOG mutant strains validated with an acquired-stress defect after other mild stresses (although the *hog1Δ* strain had a general recovery defect, perhaps due to its separate role in cell-cycle progression [51]–[55] (Table 1)). Thus, Hog1 components are required for acquisition of H_2_O_2_ tolerance if NaCl is the mild treatment but not after pretreatments that do not activate the pathway.

This explanation also holds for other pretreatments. The transcription factor Hsf1p, a critical regulator of the heat-shock response that plays an overlapping role with Msn2p [56], [57], was required for full acquisition of H_2_O_2_ tolerance following heat, but not NaCl or DTT, pretreatments. Interestingly, several regulators not previously known to respond to DTT exposure were required after this pretreatment. These included RTG transcriptional regulators (*RTG1*, *RTG2*, *RTG3*) and members of the Snf1p signaling system (*GAL83*, *STD1*, and *SNF3*) that respond to mitochondria-to-nucleus retrograde signaling and nutrient availability, respectively [58], [59]. This suggests that additional, novel regulators of the primary responses are likely being uncovered.

### Alternative lines of H_2_O_2_ defense are mobilized after each pretreatment

Although different upstream regulators were involved in each mild-stress response, we wondered if the same downstream effectors might be universally required for subsequent H_2_O_2_ tolerance. We focused on the cytosolic catalase Ctt1p, which reduces H_2_O_2_ to water and oxygen, as an obvious mechanism for detoxifying H_2_O_2_. *CTT1* was the most important gene for acquiring H_2_O_2_ resistance after mild NaCl treatment (Figure S1). Importantly, cells lacking *CTT1* had no observable sensitivity to H_2_O_2_ in the absence of pretreatment, consistent with the low basal expression of this gene ([60] and S. Haroon and APG, data not shown).

Somewhat surprisingly, *CTT1* was not universally required for acquisition of H_2_O_2_ tolerance: although the gene was critical if NaCl was the mild stressor, *CTT1* was completely dispensable after heat shock or DTT pretreatments (Figure S2). Instead, both heat shock and mild DTT treatments required the glutathione system for acquisition of H_2_O_2_ tolerance. Glutathione peroxidases provide an independent mode of H_2_O_2_ reduction that is coupled to glutathione oxidation [5]. Deletion of either of the glutathione peroxidases *GPX1* or *GPX2* did not result in an acquired stress defect (likely due to their known functional redundancy [61]). However, deletion of genes involved in glutathione metabolism, including *GSH1* that encodes the first step of glutathione synthesis and the glutathione reductase Glr1p that recycles the oxidized peptide, produced a defect after heat shock or DTT pretreatments but not NaCl. Thus, cells appear to rely on different modes of H_2_O_2_ detoxification after NaCl *versus* heat or DTT pretreatments.

We wondered why cells would utilize different detoxification mechanisms for different pretreatments. At least part of the answer lies in the gene-expression response. Although *CTT1* transcript was induced by all three mild stresses (albeit to different levels), Ctt1 protein accumulated to significant levels only after NaCl treatment (Figure S3). Neither Gsh1p nor Glr1p increased in abundance after any treatment (data not shown). However, glutathione peroxidases did increase under different conditions: Gpx2p was induced nearly 2.5-fold in response to DTT but only marginally (1.3-fold) after heat or NaCl exposure (Figure S3C). We were unable to measure Gpx1p levels by Western, although *GPX1* transcript increased after heat and NaCl treatments (data not shown and Figure S3A). These results show that the differential requirement for *CTT1* and genes involved in glutathione metabolism correlates with the conditional induction of Ctt1p or Gpx2p. Consistent with this result, we found that a double mutant lacking *CTT1* and *GSH1* had no additional defect in acquired H_2_O_2_ tolerance compared to the single mutants (data not shown).

### Unique challenges are presented depending on the mild-severe stress combination

**Figure 3:**
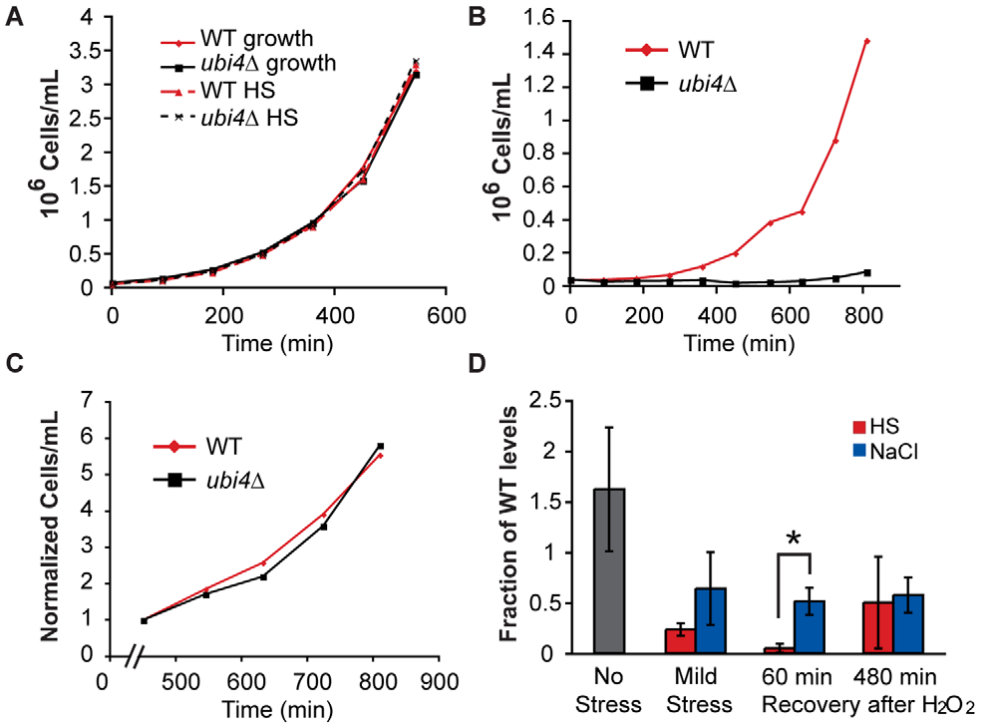
The *ubi4*? strain has a specific defect in growth recovery after H_2_O_2_ treatment. Wild-type (red) and *ubi4Δ* (black) cells were grown separately to log phase, exposed to a either a mock treatment (A, solid lines), 30–40°C heat shock (HS) for 60 min (A, dashed lines), or heat shock followed by 2 h treatment with 1.0 mM H_2_O_2_ (B). Cells were then removed from stress and monitored for growth by cell counting on a flow cytometer. (C) The growth rate normalized to cell density at 450 min after the removal from H_2_O_2_ stress is shown for wild-type (red) and *ubi4Δ* (black) cells. These growth curves are representative examples of several replicates. (D) Free ubiquitin was measured by Western analysis in wild type and *ubi4Δ* cells exposed to 1 h mild heat shock (HS, red) or NaCl (green) alone (‘Mild Stress’) and when cells were exposed to 1.0 mM H_2_O_2_ for 1 h immediately after mild stress and allowed to recover 60 or 480 min in stress-free media. Levels of free ubiquitin (normalized to an actin loading control) are shown relative to the paired wild-type sample from each of three biological replicates. A significant difference between heat- and NaCl-pretreated cells was seen 60 min after H_2_O_2_ recovery (asterisk, *p* = 0.018).

A third model for condition-specific mechanisms of acquired H_2_O_2_ tolerance is that the mode of resistance depends on the unique cellular conditions after each pretreatment. This model implies that the cell may experience H_2_O_2_ differently depending on its internal status immediately before treatment. As an example, we focused on the stress-specific poly-ubiquitin Ubi4p, which was necessary for acquired H_2_O_2_ tolerance after heat and DTT treatments but dispensable following NaCl. Ubi4p plays an important role in protein degradation and turnover in response to heat shock (reviewed in [2] and [62]). Consistent with previous observations [63], cells lacking *UBI4* were not sensitive to mild heat shock, based on viability (data not shown) or growth rate (Figure 3A). Interestingly, the *ubi4Δ* strain *was* able to acquire H_2_O_2_ resistance after heat shock, since it had wild-type viability after secondary-stress treatment (Figure S4). However, the mutant had a significant growth defect upon recovery from H_2_O_2_ stress that persisted until ∼8 h after removal from H_2_O_2_ (Figure 3B and 3C). The temporary recovery defect recapitulated the *ubi4Δ *fitness defect observed in the selection experiments.

To assess why Ubi4p was required after heat shock but not NaCl treatment, we measured free ubiquitin levels before, during, and after stress treatments (Figure 3D). Mono-ubiquitin was diminished but measurable in the *ubi4Δ* strain exposed to mild NaCl or heat shock alone. In contrast, free ubiquitin was virtually undetectable in cells treated with heat shock followed by H_2_O_2_ (Figure 3D). Mono-ubiquitin levels were again observable in the *ubi4Δ* strain 8 h after removal from H_2_O_2_, when the growth rate recovered. In contrast to the case of heat pretreatment, mono-ubiquitin was not depleted in the *ubi4Δ* strain treated with successive NaCl and H_2_O_2_. Thus, the combined effects of heat followed by H_2_O_2_ treatment require ubiquitin synthesis from the *UBI4* gene to supplant the consumed ubiquitin.

**Figure 4:**
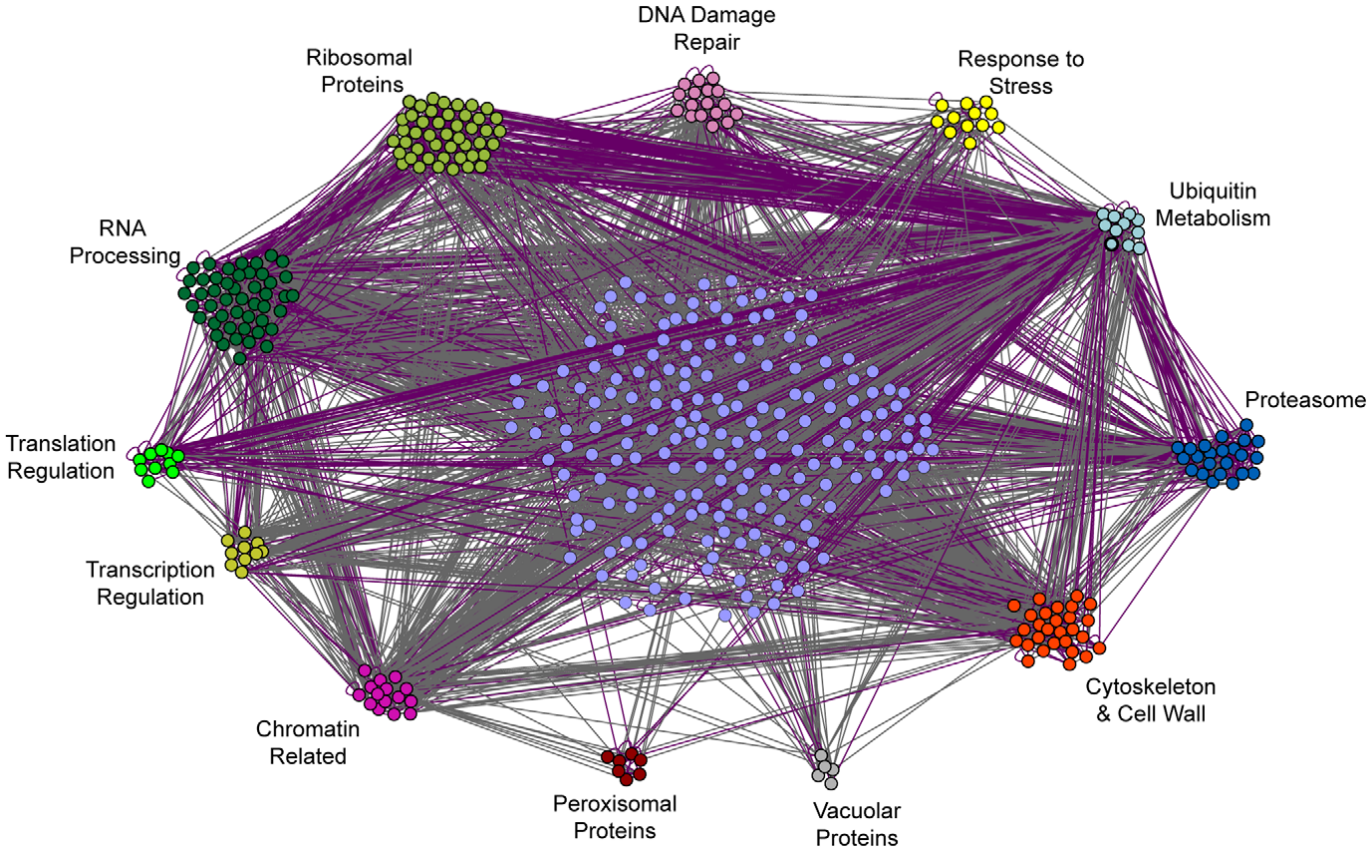
Interaction network of genes necessary for acquired H_2_O_2_ resistance following DTT pretreatment. The 497 genes necessary during DTT pretreatment were organized according to genetic (grey) and physical (purple) interactions and enriched GO-slim categories using the program GOlorize v2.4 and Cytoscape v2.4.1. The resulting network was then manually adjusted to subdivide GO-slim categories and further distinguish functional groups. Genes that could not be placed into one of the listed GO-slim categories are shown in the center of the network and were organized by GOlorize according to their interactions only.

Another possible example of context-dependent stress defense was seen when H_2_O_2_ stress followed mild DTT treatment, which invoked a large number of unique genes. To examine the connections between these genes, we constructed a network based on their genetic or physical interactions (Figure 4). The resulting network was heavily connected and pointed to a few key processes. Ribosomal proteins and proteins involved ubiquitin metabolism showed a large number of physical interactions, both within and between processes, while proteins involved in chromatin biology and actin cytoskeleton/cell wall showed the most genetic connections. This highly interconnected network demonstrates that the long list of genes important after DTT treatment can be collapsed into a smaller subset of processes.

To delineate whether the roles of these processes were related to DTT's reducing potential or to specific effects on ER function through the unfolded protein response (UPR), we repeated the selection using tunicamycin as a primary stress, to induce the UPR by blocking N-glycosylation in the ER [64]. We found that some, but not all, of the genes important after DTT treatment were also required after tunicamycin pretreatment (Table S2). Most notably, ribosomal proteins were important after both pretreatments (*p* = 6×10^−7^). Other genes required after DTT pretreatment were in fact necessary for tunicamycin survival; these were enriched for vacuolar/lysosomal transport (*p* = 8×10^−6^) and protein deubiquitination (*p* = 6×10^−6^), and included several genes linked to RNA processing. Why genes related to ribosome synthesis and protein and RNA metabolism are necessary for acquired H_2_O_2_ resistance after DTT, and to some extent tunicamycin, treatment remains unclear. However, hints from the literature suggest a connection between ER function and RNA catabolism [65]–[70]. These processes may be particularly susceptible to H_2_O_2_ attack if ER function is already disrupted (see Discussion).

## Discussion

Our results show that the genes and processes necessary to acquire resistance to the same severe stress (H_2_O_2_ in this case) are distinctly different depending on the mild stress to which cells are previously exposed. Although there were some shared processes required for pairs of pretreatments, there were surprisingly few genes required for acquisition of H_2_O_2_ tolerance after all three mild-stress treatments. Even among these shared genes, their contributions varied dramatically depending on the pretreatment. Thus, the vast majority of genes function in a condition-specific manner to produce the same end result - increased H_2_O_2_ tolerance.

We have presented three different models explaining the low degree of mechanistic overlap, including 1) condition-specific signaling, 2) use of different downstream effectors that enact the same roles, and 3) application of entirely different defense strategies based on each pretreatment. Furthermore, we note that the genes and processes involved in acquired stress resistance could function in two fundamentally different ways. Induced production and/or function of some gene products may be sufficient to boost H_2_O_2_ resistance. For example, an exogenous pulse of *CTT1* expression in the absence of stress is sufficient to increase H_2_O_2_ tolerance (S. Haroon and APG, unpublished). Alternatively, some genes and processes may be necessary, but not sufficient on their own, for acquired H_2_O_2_ resistance. Their action may instead be important to combat compounded stress, which may render some cellular processes more susceptible to oxidative attack. This model may explain the requirement for fundamental cellular processes, including RNA metabolism, ribosome biogenesis, and actin cytoskeleton, when DTT is the pretreatment. These processes are unlikely to produce H_2_O_2_ tolerance, but may instead become sensitive to H_2_O_2_ attack after DTT. Indeed, a prior study showed that ribosomal proteins are particularly prone to DTT-induced aggregation when the thioredoxin defense system is abolished [71]. Furthermore, the recent links between genes involved in RNA catabolism, P-body formation, and normal ER function [65]–[70], [72]–[74] may explain why mild stresses that trigger the UPR uniquely require these genes for subsequent stress survival.

Previous studies showed that <1% of genes required for long-term NaCl treatment showed increased expression in response to that condition [37]. Here we found that up to 24% of genes necessary for subsequent H_2_O_2_ tolerance are induced during the NaCl pretreatment. This enrichment was not true for all pretreatments, particularly DTT exposure during which most important genes showed reduced expression. Nonetheless, it suggests that gene expression is more closely correlated with, but still a relatively poor predictor of, a gene's requirement in acquired stress tolerance. The low correlation could reflect pervasive post-transcriptional regulation during mild-stress treatment. Alternatively, many genes necessary but not sufficient for acquired H_2_O_2_ tolerance may not be actively regulated in response to stress, but rather are already present at a required basal activity. Many other genes are induced during pretreatment but unnecessary for survival of either the mild stress or severe H_2_O_2_ treatment ([37], [75] and this study). It is likely that subsets of these genes (including many in the ESR) are important for acquiring resistance to other secondary stresses [31].

Beyond the mechanisms that underlie acquired stress resistance, a remaining question is its purpose. Cross-stress protection may simply be a byproduct of the overlapping effects of two stresses. For example, very high doses of NaCl can produce oxidative damage [76]; it is possible that oxidative defense mechanisms are induced during mild NaCl treatment to prepare for severe NaCl treatment rather than H_2_O_2_. Alternatively, cells may have evolved to prepare for impending stress if successive stressful environments are frequently encountered in nature, or if surviving infrequent compound stresses provides a sufficient selective advantage [31], [77], [78]. In *E. coli*, stresses that occur sequentially as bacteria travel through the gastrointestinal tract can provide cross-stress protection, and this acquired resistance is lost if cells evolve in the absence of sequential exposure [77], [78]. The role of acquired stress resistance in nature will become clearer as more is learned about the natural ecology of yeast. In the meantime, acquired stress resistance serves as an important phenotype to provide new insights into stress resistance and the complex relationship between phenotype and environment.

## Materials and Methods

### Strains and growth conditions

Strains used are shown in Table S3. We used normalized pools of the diploid homozygous non-essential yeast knockout (YKO) collection (BY4743 *MAT* ***a*** */* ***α*** *his3Δ1/his3Δ1 leu2 Δ0/leu2Δ0 lys2Δ0/LYS2 MET15/met15Δ0 ura3Δ0/ura3Δ0* background), diploid heterozygous essential YKO collection (BY4743), and DAmP yeast library (derived from BY4741/Y6683 strains (*MAT* ***a*** */* **α** *his3Δ1/his3Δ1 leu2Δ0/leu2Δ0 ura3Δ0/ura3Δ0 met15Δ0/met15Δ0 CYH2+/cyh2*) [39]. GFP-marked strain AGY0231 (*MAT* ***a*** *ura3Δ0 lys2Δ0 dORF-SWH1::Ptdh3-yEGFP-Tcyc1*) used for competition experiments was graciously provided by Barry Williams. Unless otherwise noted, cells were grown in batch culture in YPD (1% yeast extract, 2% peptone, 2% glucose) at 30°C.

### Acquired stress resistance selection experiments

Pools of the three deletion collections were grown separately for ∼7.5 generations in YPD to an optical density (OD_600_) of 0.3. The cultures were then mixed such that each strain was roughly equally represented in the resulting pools of barcodes, and an aliquot was removed as the unstressed, time 0 control sample (Sample 0). One fraction of the culture was outgrown in YPD for 10 generations (with washing and dilution in fresh YPD medium after 2, 5, and 10 generations to maintain log-phase growth). The resulting outgrown culture was collected as Sample 1 and compared to Sample 0 to identify slow-growing strains (Figure 1). A second fraction of the original culture was exposed to 0.4 mM H_2_O_2_ for two hours, centrifuged, washed, returned to fresh YPD, and outgrown 10 generations before collection as Sample 2. The remainder of the culture was exposed to one of three primary stresses, including 1 h exposure to 0.7 M NaCl, 2 h exposure to 2.5 mM DTT, or 1 h growth at 40°C after a 30°C culture was collected and resuspended in fresh 40°C medium. Following primary-stress exposure cells were centrifuged, washed, and a fraction of the culture was outgrown for 10 generations and collected as each respective Sample 3. The remaining culture was exposed to secondary stress (1.0 mM or 1.2 mM H_2_O_2_) for 2 hours, then centrifuged, washed and returned to fresh YPD medium for 10 generations outgrowth before collection as Sample 4 (1.0 mM H_2_O_2_) or Sample 4A (1.2 mM H_2_O_2_). Comparing Sample 4 to Sample 3 identified strains with a defect in acquiring resistance to severe H_2_O_2_ after mild-stress pretreatment. Most experiments were performed in at least duplicate from start to finish, with the exception of essential-gene mutants done once for NaCl and heat shock pretreatments (see Table S4). All samples were characterized by microarray analysis, and two of each experiment were also interrogated by deep sequencing (see below). Selections were also performed as above using 20 µM Tunicamycin (Sigma) for four hours as a primary stress, in biological duplicate. Fitness scores for all experiments are listed in Table S5.

### Barcode microarrays

The barcode microarrays were performed as in Pierce *et al.* 2007. Briefly, ‘up’ and ‘down’ barcodes were separately amplified from genomic DNA using common primers, and resulting PCR products were hybridized to 16K TAG4 barcode microarrays (Affymetrix part no. 511331) as previously described [79]. Each ‘up’ and ‘down’ barcode tag is represented five times on the array, for a total of 10 measurements per deletion strain. For each array, signal intensities of ‘up’ and for ‘down’ tags were averaged separately, excluding clear outliers. Quantile normalization was performed across all arrays and done separately for averaged ‘up’ and ‘down’ tag signal intensities. Following normalization, a correction factor was applied to correct for feature saturation [79], and the relative abundance of each barcoded deletion strain was then determined. Negative log_2_ ratios of strain abundance signify decreased strain fitness. Strains with positive log_2_ values, which may represent a fitness advantage, were generally not confirmed in validation assays and are not discussed further (data not shown).

### Barcode sequencing

The sequencing protocol was adapted from [80]. Barcodes were amplified from genomic DNA using primers that included the common YKO barcode amplification sequences, the Illumina anchor sequences, and multiplex indexes for sample multiplexing (sequences available in Table S6), using Herculase II Fusion DNA polymerase (Agilent). PCR products of ∼150 bp were purified using the e-Gel gel purification system and SybrGreen (Invitrogen). Two biological replicates of Samples 1, 2, 3, and 4 were sequenced for each selection.

Finished libraries were sent to the University of Wisconsin Sequencing Facility for Illumina sequencing. Briefly, quality and quantity of the finished libraries were assessed using an Agilent DNA 1000 series chip assay and QuantIT PicoGreen dsDNA Kit (Invitrogen), respectively. Each library was standardized to 10 µM, then 12 uniquely indexed upstream barcode libraries and 12 uniquely indexed downstream barcode libraries were pooled in each lane (representing ‘up’ and ‘down’ tags from 12 different Samples above). Cluster generation was performed using a standard Cluster Kit (v4) and the Illumina Cluster Station, or a standard cBot Kit (v4) and the Illumina cBot. Single-end 50 bp or 75 bp reads were collected using standard SBS kits (v4) and SCS 2.5 software, on an Illumina Genome Analyzer IIx. Images were analyzed using the standard Illumina Pipeline, version 1.5, and the sequence reads were mapped back to YKO barcode sequences using custom scripts, allowing one mismatch per 6-bp multiplex sequence and two mismatches per 20 bp ‘up’ or ‘down’ tag (discarding mismatches that did not map uniquely).

### Validation

Survival experiments were performed as in [31], except viability was scored using an EasyCyte flow cytometer (Millipore) and LIVE/DEAD Fungalite Yeast Viability Kit (Invitrogen). Briefly, cells were exposed to mild stress or mock treatment in flasks, collected by centrifugation, and resuspended in YPD. Cells were then exposed to 12 doses of H_2_O_2_ (0–5 mM) in 96-well plates for 2 hours, and incubated with dye for 30–60 min before fluorescence was scored at each H_2_O_2_ dose. Survival scores shown in the figures were based on the fraction of pretreated cells that survived each dose, minus the fraction of mock treated cells that survived that dose – a single score was then computed as the sum of those values across all doses of secondary stress [31]. *CTT1* results were also validated in an independent *ctt1Δ::URA3* strain that was then complimented with *CTT1* on a plasmid (data not shown).

GFP competition experiments were performed by competing a GFP-marked strain against either wild-type BY4741 or single-gene deletion strains from the haploid yeast deletion library (Open Biosystems). Cells were grown separately overnight to early log phase (OD_600_ 0.3) and mixed at a 1∶5 ratio of GFP-marked: unmarked cells. Mixed cultures were exposed to no stress, primary stress alone, 0.4 mM H_2_O_2_ alone, or primary stress followed by 1.0 mM H_2_O_2_ for 2 hours; cells were then washed with YPD and grown 10 generations in YPD in the absence of stress. Relative strain abundance was inferred based on the proportion of GFP-expressing cells assayed using the EasyCyte flow cytometer (Millipore) before and after outgrowth [81]. The proportion of GFP-expressing cells when mixed with a given deletion strain was compared to proportion of GFP-expressing cells mixed with wild-type BY4741; an increase in the number of GFP-marked: mutant cells, relative to the wild-type control, indicated a competition defect in the deletion strain of interest.

### Western blots

BY4741 and *ubi4Δ* cells were grown at least 7 generations to early log phase and a sample of each culture was collected for an unstressed control. The culture was exposed to 30–40°C heat shock or 0.7 M NaCl for one hour, then washed and exposed to 1.0 mM H_2_O_2_ for 2 hours, and washed and outgrown in YPD. Cell samples were collected before and after pretreatment and at 1 h and 8 h during YPD outgrowth.

Whole-cell lysate was assayed by Western analysis with the following primary antibodies: polyclonal rabbit anti-ubiquitin (kindly provided by R. Vierstra), monoclonal mouse anti-FLAG (F3165, Sigma), polyclonal rabbit anti-TAP (CAB1001, Open Biosystems), or monoclonal mouse anti-actin (MAB1501; Millipore,Billerica, MA). Secondary antibodies included LiCor (Lincoln, NE) IRDye 680LT goat anti-rabbit (926–68021) or goat anti-mouse (926–32210) fluorescent antibodies. Blots were visualized and analyzed using an Odyssey Infrared Imaging System v3.0.21. Free ubiquitin, FLAG-Ctt1p, or Gpx2-TAPp were normalized to actin in each lane.

### Quantitative PCR

Quantitative PCR was done as previously described [82] using iQSYBR Green Supermix (Bio-Rad, Hercules, CA) on a MyiQ2 Bio-Rad Cycler. Primers spanned a 3' 100–200 bp region of each ORF. Cycle numbers were normalized ERV25 mRNA as an internal control unaffected by stress.

### Data analysis

For each strain, fitness after a particular treatment was taken as the log_2_ change in strain abundance between each Sample and its corresponding control (see Figure 1). Strains were identified as defective in acquired stress resistance if they met the following criteria in the microarray and/or sequencing experiments: 1) Strains displayed a fitness defect in response to 1.0 mM H_2_O_2_ following primary treatments (e.g. Sample 4 compared to Sample 3) that was at least 1 standard deviation from the mean of all strains. 2) The fitness defect following primary-stress treatment alone (Sample 3 versus Sample 1) was <1 standard deviation from the mean of all strains. 3) The fitness defect in response to 0.4 mM H_2_O_2_ (Sample 2 versus Sample 1) was less than the defect in 1.0 mM (or 1.2 mM) H_2_O_2_. 4) These criteria were true in at least two replicates. These stringent lists were expanded by manually adding strains whose fitness phenotypes were highly correlated with identified mutants. For libraries with only one replicate (for example, the heterozygous deletion collection used in the NaCl selection), identified strains were required to meet the stringent criteria for both 1.0 mM and 1.2 mM H_2_O_2_ doses or in corresponding mutants from multiple libraries (e.g. a significant defect in both the heterozygous-gene deletion strain and DAmP strain).

Clustering was done in Cluster 3.0 (http://bonsai.hgc.jp/~mdehoon/software/cluster/software.htm) using hierarchical clustering and uncentered Pearson correlation as the metric [83]. Enrichment of gene functional categories was performed using the hypergeometric distribution in Excel or the program Funspec [84] with Bonferroni-corrected p-values <0.01 taken as significant. Network graphs were constructed using Cytoscape 2.8 [85]. Genetic and physical interactions were downloaded from BioGRID release 3.0.66 [86]. Enrichment of genetic or physical interactions, compared to random chance, was determined for 1000 randomly sampled networks with the same number of genes and assessing the number of trials with equal or greater number of total pairwise connections to the observed networks. Genes with defects in acquired stress resistance were defined as induced or repressed during pretreatments if the average (n> = 3) expression change was greater than 1.5X higher or lower than unstressed cells 45 min after 0.7 M NaCl or 15 min after a 30–37°C heat shock [31], or 90 min after 2.5 mM DTT (S. Topper and APG, unpublished).

## Supporting Information

Figure S1 The HOG pathway provides a direct link between osmotic stress and acquired H_2_O_2_ resistance. The average and standard deviation (n = 3) of survival scores are shown for cells treated with 0–5 mM H_2_O_2_ following pretreatment with 0.7 M NaCl, as described in Materials and Methods. The survival score was calculated based on the percent viability at each of 11 doses of severe H_2_O_2_, minus the percent viability of mock-treated cells, summed over all doses to produce a single store.
(PDF)

Figure S2 *CTT1* is necessary for acquiring H_2_O_2_ tolerance following NaCl but not heat shock or DTT pretreatments. The average and standard deviation of survival scores is shown as assayed in Figure S1, for wild type (red) and the *ctt1Δ* strain (yellow). Data represent three biological replicates for NaCl or duplicate experiments for heat shock and DTT pretreatments.
(PDF)

Figure S3 Differential expression of H_2_O_2_ detoxification genes. (A) The log_2_ change in abundance of *CTT1*, *GPX1*, and *GPX2* mRNA is shown at the peak of each response, including 120 min after 2.5 mM DTT treatment, 10 min after 30–40C heat shock, or 30 min after 0.7 M NaCl, as measured by qPCR. (B) Expression of genomically expressed FLAG-tagged Ctt1p or Act1p as a loading control under the following conditions: 1) no stress, 2) 120 min 2.5 mM DTT, 3) no stress, 4) 60 min after 30–40C heat shock, 5) no stress, 6) 60 min after 0.7 M NaCl. The results show a significant increase in FLAG-Ctt1p after NaCl, and a barely detectible band after heat shock but not DTT treatment. Comparing Act1p-normalized FLAG-Ctt1p after NaCl versus heat shock revealed ∼7X more FLAG-Ctt1p induced after NaCl treatment. (C) The log_2_ change in abundance of C-terminally TAP-tagged Gpx2p was measured by quantitative Western. Gpx2-TAPp was normalized to Act1p as a loading control, and the fold change was calculated relative to unstressed Gpx2-TAPp levels measured for each condition. Error bars represent standard deviation of 3 or 5 biological replicates for qPCR and Western analysis, respectively. We were unable to measure Gpx1-TAPp by Western analysis. Notably, changes in protein abundance (B and C) did not correlate well with changes in mRNA abundance (A), making interpretation of *GPX1* transcript induction after NaCl treatment difficult to interpret.
(PDF)

Figure S4 Cells lacking *UBI4* can acquire H_2_O_2_ resistance but have a fitness defect during recovery. (A) Average and standard deviation (n = 4) of H_2_O_2_ survival scores following pretreatment with 30–40°C heat shock is shown, as assayed in Figure S1. (B) Competitive fitness of the *ubi4Δ* or isogentic wild-type cells was measured by competing each strain against a GFP-expressing strain and scoring relative strain abundances after 10 generations of growth (‘Fitness Defect’ relative to GFP strain). Mixed cultures were exposed to no stress (Growth Alone), a 30–40°C heat shock (HS), or heat shock followed by severe (1.0 mM) H_2_O_2_ (HS + H_2_O_2_). Cells were then removed from stress and outgrown 10 generations in YPD before relative strain abundances were measured. Error bars represent one standard deviation based on 4 biological replicates (A) or duplicate experiments (B). (C) Growth recovery in YPD after exposure to mild (0.7 M) NaCl followed by severe (1.0 mM) H_2_O_2_, for wild type (black) and *ubi4*? (red) cells. The graph shown is a representative of 3 replicates.
(PDF)

Table S1 Strains with a fitness defect after 2 hours 0.4 mM H_2_O_2_ treatment. Strains with significant fitness defects were identified from six sequenced replicates comparing Sample 1 to Sample 2 (see Figure 1), with a cutoff of q<0.05.
(XLS)

Table S2 Mutant strains with defects in acquiring H2O2 tolerance after each mild-stress pretreatment. Mutants were identified as described in Materials and Methods.
(XLS)

Table S3 Strains used in this study.
(PDF)

Table S4 Number of biological replicates performed for each library. The number of selections performed with each library is shown. All pools were interrogated by microarray analysis; the number of biological replicates sequenced is shown in parentheses.
(PDF)

Table S5 Measured fitness defects. Each value is the Log2 comparison of the samples indicated in the column header (for example, Sample1 vs Sample0, see Figure 1). Comparisons measured by microarray are labeled "Array". Comparisons measured by deep sequencing are labeled either "UP" or "DN", corresponding to the values from the unique tag 5′ (UP) or 3′ (DN) of each gene. Missing data is denoted as "NA". The Homozygous and Heterozygous data are presented together (“Hom-Het Compilation”). The DaMP array data was normalized separately from the corresponding Homozygous and Heterozygous samples and is therefore presented in a separate tab (“DaMP Compilation”). Biological replicates are indicated by the replicate number (for example, 'NaCl2' denotes the second replicate of the NaCl experiment).
(XLS)

Table S6 Primer sequences used for barcode sequencing. U1,U2 and D1,D2 sequences correspond to common sequences flanking the unique “up” tag 5′ (U) or “down” tag 3′ (D) of each gene knock-out cassette in the yeast YKO libraries. Multiplexing primers used for barcode sequencing (including the U and D sequences) are shown.
(XLS)
