# Supplementary figures and images for: Multiple Means to the Same End: The Genetic Basis of Acquired Stress Resistance in Yeast
Source: PLoS Genet. 2011 Nov 10;7(11):e1002353. doi: 10.1371/journal.pgen.1002353 (PMC3213159; doi:10.1371/journal.pgen.1002353)

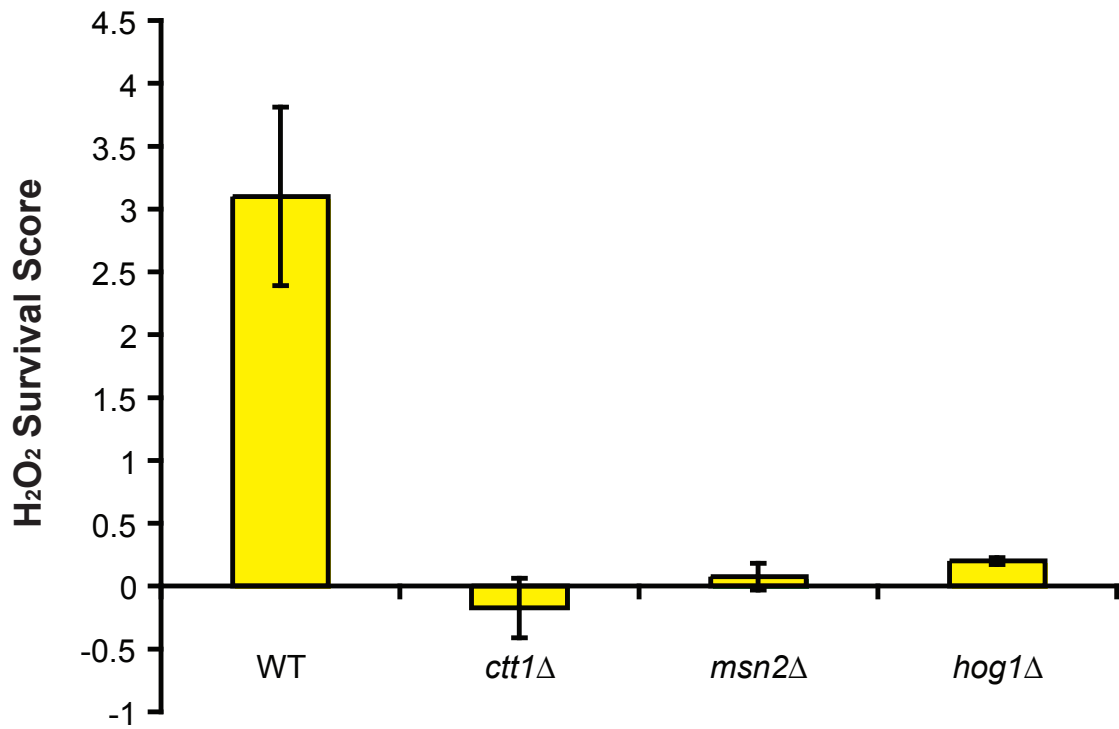

Supplement: Figure S1 — The HOG pathway provides a direct link between osmotic stress and acquired H2O2 resistance. The average and standard deviation (n = 3) of survival scores are shown for cells treated with 0–5 mM H2O2 following pretreatment with 0.7 M NaCl, as described in Materials and Methods. The survival score was calculated based on the percent viability at each of 11 doses of severe H2O2, minus the percent viability of mock-treated cells, summed over all doses to produce a single store. (PDF) [file pgen.1002353.s001.pdf]

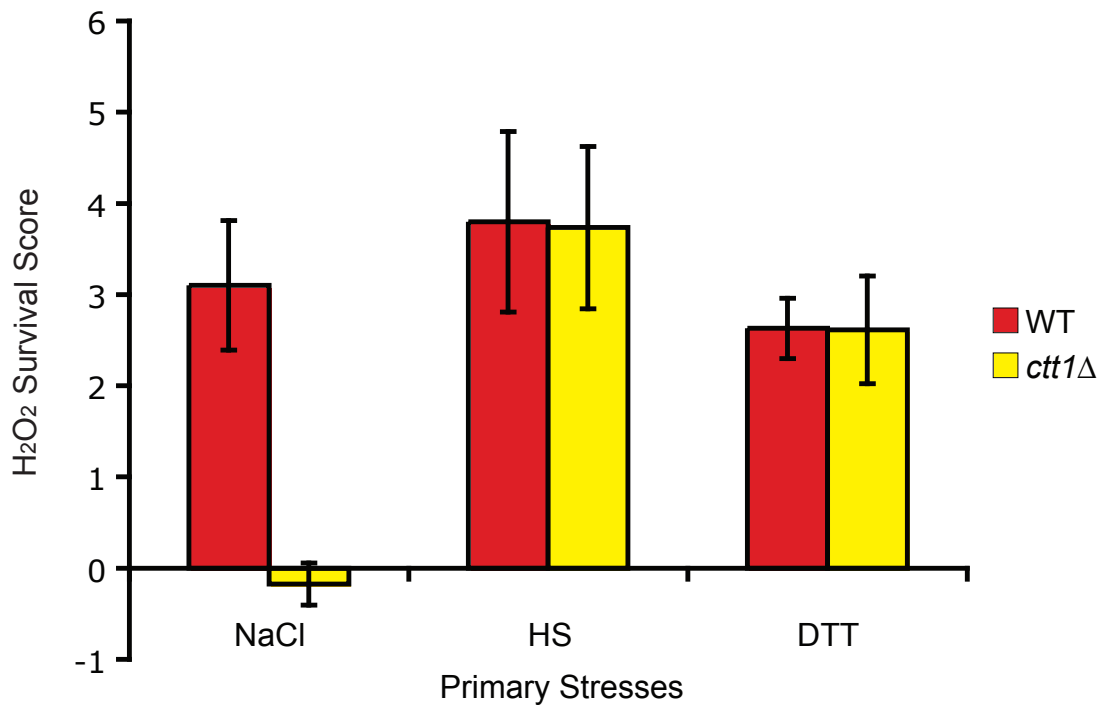

Supplement: Figure S2 — CTT1 is necessary for acquiring H2O2 tolerance following NaCl but not heat shock or DTT pretreatments. The average and standard deviation of survival scores is shown as assayed in Figure S1, for wild type (red) and the ctt1Δ strain (yellow). Data represent three biological replicates for NaCl or duplicate experiments for heat shock and DTT pretreatments. (PDF) [file pgen.1002353.s002.pdf]

A

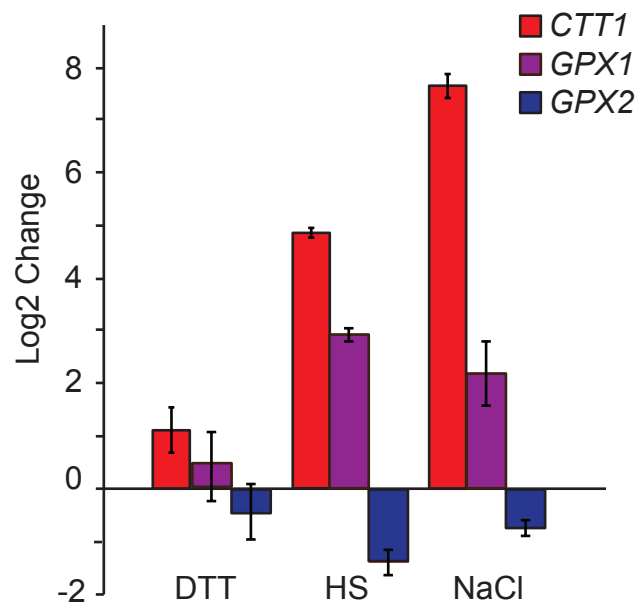

B

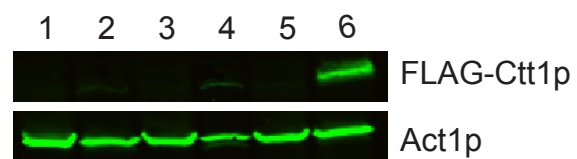

C

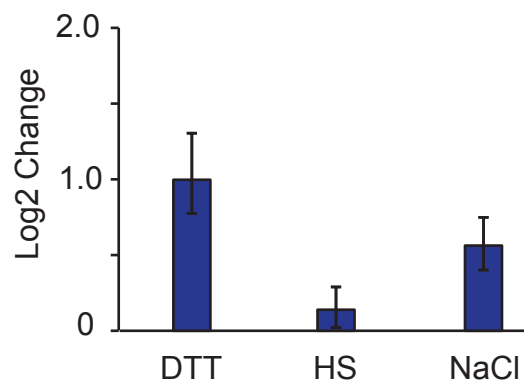

Supplement: Figure S3 — Differential expression of H2O2 detoxification genes. (A) The log2 change in abundance of CTT1, GPX1, and GPX2 mRNA is shown at the peak of each response, including 120 min after 2.5 mM DTT treatment, 10 min after 30–40C heat shock, or 30 min after 0.7 M NaCl, as measured by qPCR. (B) Expression of genomically expressed FLAG-tagged Ctt1p or Act1p as a loading control under the following conditions: 1) no stress, 2) 120 min 2.5 mM DTT, 3) no stress, 4) 60 min after 30–40C heat shock, 5) no stress, 6) 60 min after 0.7 M NaCl. The results show a significant increase in FLAG-Ctt1p after NaCl, and a barely detectible band after heat shock but not DTT treatment. Comparing Act1p-normalized FLAG-Ctt1p after NaCl versus heat shock revealed ∼7X more FLAG-Ctt1p induced after NaCl treatment. (C) The log2 change in abundance of C-terminally TAP-tagged Gpx2p was measured by quantitative Western. Gpx2-TAPp was normalized to Act1p as a loading control, and the fold change was calculated relative to unstressed Gpx2-TAPp levels measured for each condition. Error bars represent standard deviation of 3 or 5 biological replicates for qPCR and Western analysis, respectively. We were unable to measure Gpx1-TAPp by Western analysis. Notably, changes in protein abundance (B and C) did not correlate well with changes in mRNA abundance (A), making interpretation of GPX1 transcript induction after NaCl treatment difficult to interpret. (PDF) [file pgen.1002353.s003.pdf]

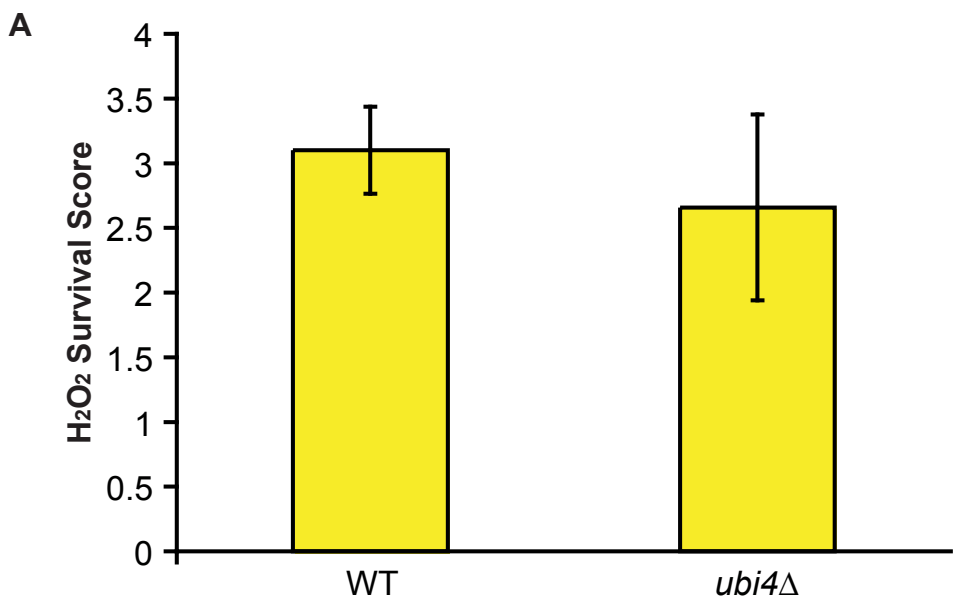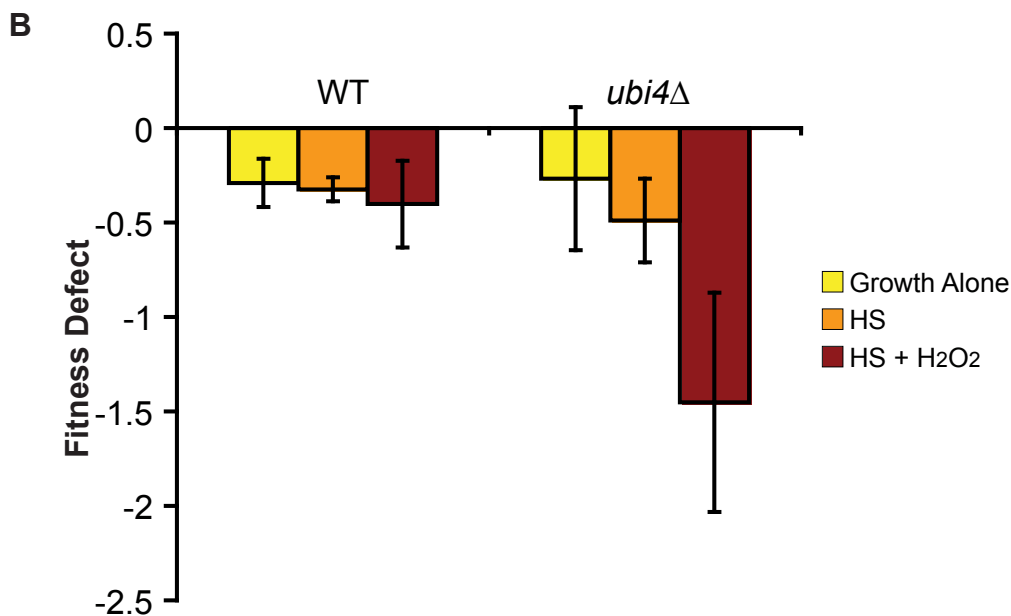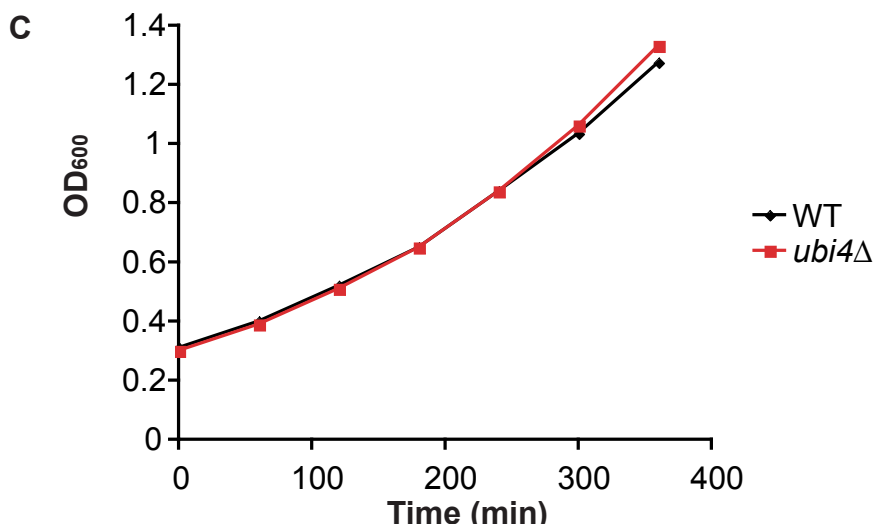

Supplement: Figure S4 — Cells lacking UBI4 can acquire H2O2 resistance but have a fitness defect during recovery. (A) Average and standard deviation (n = 4) of H2O2 survival scores following pretreatment with 30–40°C heat shock is shown, as assayed in Figure S1. (B) Competitive fitness of the ubi4Δ or isogentic wild-type cells was measured by competing each strain against a GFP-expressing strain and scoring relative strain abundances after 10 generations of growth (‘Fitness Defect’ relative to GFP strain). Mixed cultures were exposed to no stress (Growth Alone), a 30–40°C heat shock (HS), or heat shock followed by severe (1.0 mM) H2O2 (HS + H2O2). Cells were then removed from stress and outgrown 10 generations in YPD before relative strain abundances were measured. Error bars represent one standard deviation based on 4 biological replicates (A) or duplicate experiments (B). (C) Growth recovery in YPD after exposure to mild (0.7 M) NaCl followed by severe (1.0 mM) H2O2, for wild type (black) and ubi4? (red) cells. The graph shown is a representative of 3 replicates. (PDF) [file pgen.1002353.s004.pdf]
