# Supplementary material for: Multiple Means to the Same End: The Genetic Basis of Acquired Stress Resistance in Yeast
Source: PLoS Genet. 2011 Nov 10;7(11):e1002353. doi: 10.1371/journal.pgen.1002353 (PMC3213159; doi:10.1371/journal.pgen.1002353)
Supplement: Table S3 — Strains used in this study. (PDF) [file pgen.1002353.s007.pdf]

Strain	Genotype
BY4741	Mat a *his3Δ1 leu2Δ0 met15Δ0 ura3Δ0*
AGY0231	Mat a *ura3Δ0 lys2Δ0 dORF-SWH1::Ptdh3-yEGFP-Tcyc1*
YKO7117	Mat a *his3Δ1 leu2Δ0 met15Δ0 ura3Δ0 msn2::KanMX*
YKO2724	Mat a *his3Δ1 leu2Δ0 met15Δ0 ura3Δ0 hog1::KanMX*
YKO0615	Mat a *his3Δ1 leu2Δ0 met15Δ0 ura3Δ0 sub1::KanMX*
YKO4752	Mat a *his3Δ1 leu2Δ0 met15Δ0 ura3Δ0 ygr122w::KanMX*
YKO1657	Mat a *his3Δ1 leu2Δ0 met15Δ0 ura3Δ0 pde2::KanMX*
YKO4010	Mat a *his3Δ1 leu2Δ0 met15Δ0 ura3Δ0 pph3::KanMX*
YKO0304	Mat a *his3Δ1 leu2Δ0 met15Δ0 ura3Δ0 npr2::KanMX*
YKO1137	Mat a *his3Δ1 leu2Δ0 met15Δ0 ura3Δ0 mck1::KanMX*
YKO7281	Mat a *his3Δ1 leu2Δ0 met15Δ0 ura3Δ0 rim15::KanMX*
YKO0397	Mat a *his3Δ1 leu2Δ0 met15Δ0 ura3Δ0 swc3::KanMX*
YKO3371	Mat a *his3Δ1 leu2Δ0 met15Δ0 ura3Δ0 swc5::KanMX*
YKO0940	Mat a *his3Δ1 leu2Δ0 met15Δ0 ura3Δ0 npr3::KanMX*
YKO0351	Mat a *his3Δ1 leu2Δ0 met15Δ0 ura3Δ0 gpb2::KanMX*
YKO1261	Mat a *his3Δ1 leu2Δ0 met15Δ0 ura3Δ0 tpk1::KanMX*
YKO0936	Mat a *his3Δ1 leu2Δ0 met15Δ0 ura3Δ0 rim101::KanMX*
YKO1819	Mat a *his3Δ1 leu2Δ0 met15Δ0 ura3Δ0 whi2::KanMX*
YKO1527	Mat a *his3Δ1 leu2Δ0 met15Δ0 ura3Δ0 ubi4::KanMX*
YSC1178-7499403	Mat a *his3Δ1 leu2Δ0 met15Δ0 ura3Δ0 GPX2-TAP-HIS3*
YKO7097	Mat a *his3Δ1 leu2Δ0 met15Δ0 ura3Δ0 gsh1::KanMX*
YKO2737	Mat a *his3Δ1 leu2Δ0 met15Δ0 ura3Δ0 glr1::KanMX*
AGY612	Mat a *his3Δ1 leu2Δ0 met15Δ0 ura3Δ0 ctt1::URA3 gsh1::KanMX*
AGY633	*Mat a his3Δ1 leu2Δ0 met15Δ0 ura3Δ0 ctt1::FLAG-CTT1*
