# Supplementary material for: Multiple Means to the Same End: The Genetic Basis of Acquired Stress Resistance in Yeast
Source: PLoS Genet. 2011 Nov 10;7(11):e1002353. doi: 10.1371/journal.pgen.1002353 (PMC3213159; doi:10.1371/journal.pgen.1002353)
Supplement: Table S4 — Number of biological replicates performed for each library. The number of selections performed with each library is shown. All pools were interrogated by microarray analysis; the number of biological replicates sequenced is shown in parentheses. (PDF) [file pgen.1002353.s008.pdf]

	Deletion Library		
Mild Stress	**Homozygous Diploid**	**Heterozygous Diploid**	**DAmP alleles**
NaCl Primary	3 (2)	1 (1)	1 (1)
HS Primary	3 (2)	1 (1)	1 (1)
DTT Primary	2 (2)	2 (2)	2 (2)

Table S4: Number of biological replicates performed for each library. The number of selections performed with each library is shown. All pools were interrogated by microarray analysis; the number of biological replicates sequenced is shown in parentheses.
